# Supplementary material for: The Saint-Leonard Urban Glaciotectonic Cave Harbors Rich and Diverse Planktonic and Sedimentary Microbial Communities
Source: Microorganisms. 2024 Aug 29;12(9):1791. doi: 10.3390/microorganisms12091791 (PMC11434022; doi:10.3390/microorganisms12091791)
Supplement: Supplementary file 1 [file microorganisms-12-01791-s001.zip › microorganisms-3156725-supplementary.pdf]

microorganisms

MDPI

Table S1. Comparison of alpha-diversity indices using Kruskal-Wallis tests and multiple comparisons, based on Shannon indices, ASV richness, and evenness, for the Bacteria domain. SS, surface soils; S, sediments; W1, water 0.1 μm; W2, water 0.2 μm.

Kruskal-Wallis	Chi-squared	df	Pvalue
**Shannon**	6.7253	3	0.08119
**ASV richness**	10.583	3	0.01421
**Evenness**	19.229	3	0.0002451
**Comparison**	**Z**	**Pvalue**	
**ASV richness**			
SS-S	1.6363656	0.101763099	
SS-W1	-1.6038758	0.108741432	
SS-W2	1.2517153	0.210673621	
S-W1	0.2104939	0.833282193	
S-W2	2.6323128	0.008480573	
W1-W2	2.7213442	0.006501702	
**Evenness**			
SS-S	-2.801236	0.005090728	
SS-W1	4.0149309	0.00005946321	
SS-W2	1.1954584	0.231908	
S-W1	0.7016464	0.4828997	
S-W2	-1.7497138	0.08016771	
W1-W2	-2.7853074	0.005347699	

Table S2. Comparison of alpha-diversity indices using Kruskal-Wallis tests and multiple comparisons, based on Shannon indices, ASV richness, and evenness, for the Archaea domain. SS, surface soils; S, sediments; W1, water 0.1 μm; W2, water 0.2 μm.

Kruskal-Wallis	Chi-squared	df	Pvalue
**Shannon**	17.186	3	0.000647
**ASV richness**	14.224	3	0.002615
**Evenness**	13.564	3	0.003563
**Comparison**	**Z**	**Pvalue**	
**Shannon**			
SS-S	0.2855201	0.7752456918	
SS-W1	-3.4575918	0.0005450266	
SS-W2	-2.9007254	0.0037229993	
S-W1	-2.8463257	0.0044226932	
S-W2	-2.2572347	0.0239934104	
W1-W2	0.9246033	0.3551722841	
**ASV richness**			
SS-S	0.4379057	0.6614546366	
SS-W1	-2.4479673	0.0143664679	
SS-W2	-3.3387796	0.0008414731	
S-W1	-1.7516228	0.0798386829	
S-W2	-2.5126050	0.0119843421	
W1-W2	-0.7984038	0.4246361927	
**Evenness**			
SS-S	0.3426241	0.7318812357	
SS-W1	-2.2132670	0.0268792447	
SS-W2	1.3061562	0.1914994525	
S-W1	-2.3222303	0.0202205376	
S-W2	0.8065234	0.4199411354	
W1-W2	3.6029719	0.0003145996	

Table S3. Comparison of alpha-diversity indices using Kruskal-Wallis tests and multiple comparisons, based on Shannon indices, ASV richness, and evenness, for the Eukaryote domain. SS, surface soils; S, sediments; W2, water 0.2 μm.

Kruskal-Wallis.	Chi-squared	df	Pvalue
**Shannon**	9.9062	3	0.01938
**ASV richness**	18.28	3	0.0003851
**Evenness**	3.0876	3	0.3783
**Comparison**	**Z**	**Pvalue**	
**Shannon**			
SS-S	-1.2409674	0.214617805	
SS-W2	-1.096097	0.27303634	
S-W2	-2.175722	0.029576054	
**ASV richness**			
SS-S	-1.805043	0.0710678847	
SS-W2	-2.116601	0.034293721	
S-W2	-3.610087	0.0003060945	

Table S4. PERMANOVA analyses based on a Bray-Curtis dissimilarity matrix using habitat (surface soils, cave sediment, and cave water 0.1 and 0.2 μm) as an environmental variable, for the Bacteria and Archaea domains. The habitats were surface soils, cave sediment, and cave water 0.2 μm for the Eukaryote domain.

	Df	SumofSqs	R2	F	Pr(>F)
**BACTERIA**					
Habitat	3	5.2691	0.51285	7.3694	0.001
Residual	21	5.0049	0.48715		
Total	24	10.2740	1.00000		
**ARCHAEA**					
Habitat	3	5.4621	0.63285	10.916	0.001
Residual	19	3.1689	0.36715		
Total	22	8.6310	1.00000		
**EUKARYOTE**					
Habitat	2	2.7245	0.39383	4.8727	0.001
Residual	15	4.1935	0.60617		
Total	17	6.9180	1.00000		

Table S5. AMOVA analyses based on a Bray-Curtis dissimilarity matrix using habitat (surface soils, cave sediment, and cave water 0.1 and 0.2 µm) as an environmental variable, for the Bacteria. SS, surface soils; S, sediments; W1, water 0.1 µm; W2, water 0.2 µm; SW, surface water.

	Among	Within	Total	
**All**				
SS	5.26905	5.00491	10.274	
dF	3	21	24	
MS	1.75635	0.238329		
Fs				7.36944
p-value				<0.001
**SS-S**				
SS	1.23831	3.29541	4.53373	
dF	1	10	11	
MS	1.23831	0.329541		
Fs				3.75768
p-value				0.004
**SS-W1**				
SS	1.76503	3.46331	5.22834	
dF	1	12	13	
MS	1.76503	0.288609		
Fs				6.11562
p-value				<0.001
**SS-W2**				
SS	2.29581	2.94126	5.23707	
dF	1	13	14	
MS	2.29581	0.226251		
Fs				10.1472
p-value				<0.001
**S-W1**				
SS	1.29608	2.06365	3.35974	
dF	1	8	9	
MS	1.29608	0.257957		
Fs				5.02442
p-value				0.005
**S-W2**				
SS	1.69644	1.5416	3.23804	
dF	1	9	10	
MS	1.69644	0.171289		
Fs				9.904
p-value				<0.001
**W1-W2**				
SS	2.00749	1.7095	3.71699	
dF	1	11	12	
MS	2.00749	0.155409		
Fs				12.9175
p-value				0.001

Table S6. AMOVA analyses based on a Bray-Curtis dissimilarity matrix using habitat (surface soils, cave sediment, and cave water 0.1 and 0.2 µm) as an environmental variable, for the Archaea. SS, surface soils; S, sediments; W1, water 0.1 µm; W2, water 0.2 µm; SW, surface water.

	Among	Within	Total	
**All**				
SS	5.46208	3.16889	8.63098	
dF	3	19	22	
MS	1.82069	0.166784		
Fs				10.9165
p-value				0.001
**SS-S**				
SS	1.37125	2.16192	3.53316	
dF	1	8	9	
MS	1.37125	0.27024		
Fs				5.0742
p-value				0.003
**SS-W1**				
SS	1.86672	2.25271	4.11942	
dF	1	10	11	
MS	1.86672	0.225271		
Fs				8.28655
p-value				0.001
**SS-W2**				
SS	2.30972	1.57305	3.88277	
dF	1	11	12	
MS	2.30972	0.143004		
Fs				16.1514
p-value				0.003
**S-W1**				
SS	1.55459	1.59585	3.15043	
dF	1	8	9	
MS	1.55459	0.199481		
Fs				7.79315
p-value				0.004
**S-W2**				
SS	1.90638	0.916186	2.82256	
dF	1	9	10	
MS	1.90638		0.101798	
Fs				18.727
p-value				0.004
**W1-W2**				
SS	1.76711	1.00698	2.77409	
dF	1	11	12	
MS	1.76711	0.0915434		
Fs				19.3036
p-value				0.001

Table S7. AMOVA analyses based on a Bray-Curtis dissimilarity matrix using habitat (surface soils, cave sediment, and cave water 0.2 µm) as an environmental variable, for the Eukaryote. SS, surface soils; S, sediments; W2, water 0.2 µm; SW, surface water.

	Among	Within	Total	
**All**				
SS	2.72448	4.1935	6.91798	
dF	2	15	17	
MS	1.36224	0.279567		
Fs				4.87268
p-value				<0.001
**SS-S**				
SS	0.796042	2.90643	3.70248	
dF	1	9	10	
MS	0.796042	0.322937		
Fs				2.465
p-value				0.003
**SS-W2**				
SS	1.86592	3.01892	4.88484	
dF	1	12	13	
MS	1.86592	0.251577		
Fs				7.41693
p-value				<0.001
**S-W2**				
SS	1.28738	2.46164		3.74903
dF	1	9	10	
MS	1.28738	0.273516		
Fs				4.70678
p-value				0.002

Table S8. HOMOVA analyses based on a Bray-Curtis dissimilarity matrix comparing cave water 0.2 (W2) and 0.1 (W1) µm samples, for the Bacteria and Archaea domains.

	BValue	P-value	SSwithin/(N-1)_values	
			**W1**	**W2**
Bacteria	0.823698	0.007	0.223155	0.0989535
Archaea	3.85496	0.004	0.168664	0.0272763

Table S9. db-RDA analyses based on a Bray-Curtis dissimilarity matrix for the surface soil/cave sediment communities as well as the cave water 0.2 μm communities for all 3 domains, and the cave water 0.1 μm communities for the Bacteria and Archaea domains, and environmental variables measured in each sample. tot, total; org, organic; inorg, inorganic; SoS, SumOfSqs.

	BACTERIA				ARCHAEA				EUKARYOTE			
	Df	SoS	F	Pr(>F)	Df	SoS	F	Pr(>F)	Df	SoS	F	Pr(>F)
**Soil/Sediments**												
pH	1	0.69251	2.1159	0.003	1	1.03584	3.9466	0.008	1	0.70173	2.4631	0.012
Ctot	1	0.40209	1.2285	0.213	1	0.29826	1.1364	0.325	1	0.24526	0.8609	0.619
Corg	1	0.38685	1.1820	0.255	1	0.24336	0.9272	0.484	1	0.30758	1.0796	0.332
Cinorg	1	0.30256	0.9244	0.547	1	0.23710	0.9034	0.539	1	0.34873	1.2240	0.227
Ntot	1	0.55689	1.7015	0.031	1	0.34423	1.3115	0.253	1	0.27023	0.9485	0.536
$\delta^{13}C$	1	0.48418	1.4793	0.082	1	0.31459	1.1986	0.292	1	0.36436	1.2789	0.189
Residual	3	0.98189			2	0.52492			2	0.56980		
**Water 0.2 μm**												
DIC	1	0.15371	1.2129	0.086	1	0.083487	1.1955	0.035	1	0.225233	1.7533	0.142
DOC	1	0.17432	1.3756	0.014	1	0.074546	1.0675	0.286	1	0.092212	0.7178	0.688
NHx	1	0.12353	0.9748	0.508	1	0.065149	0.9329	0.668	1	0.094777	0.7378	0.658
NO_3^-	1	0.12813	1.0111	0.435	1	0.088699	1.2701	0.023	1	0.078628	0.6121	0.789
Residual	2	0.25345			2	0.139670			2	0.256930		
**Water 0.1 μm**												
DIC	1	0.28232	0.7755	0.8750	1	0.21246	1.1640	0.4708				
DOC	1	0.32200	0.8845	0.7042	1	0.34974	1.9161	0.0875				
NHx	1	0.29307	0.8050	0.7931	1	0.18416	1.0089	0.4944				
NO_3^-	1	0.26997	0.7416	0.9722	1	0.22242	1.2185	0.3361				
Residual	1	0.36405			1	0.18253						

Supplemental Material Figure Legends

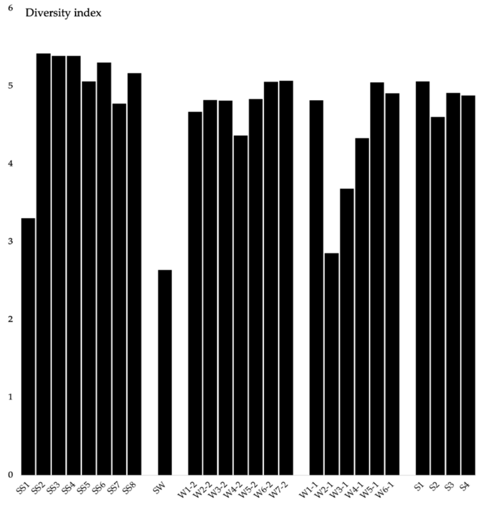

(a)

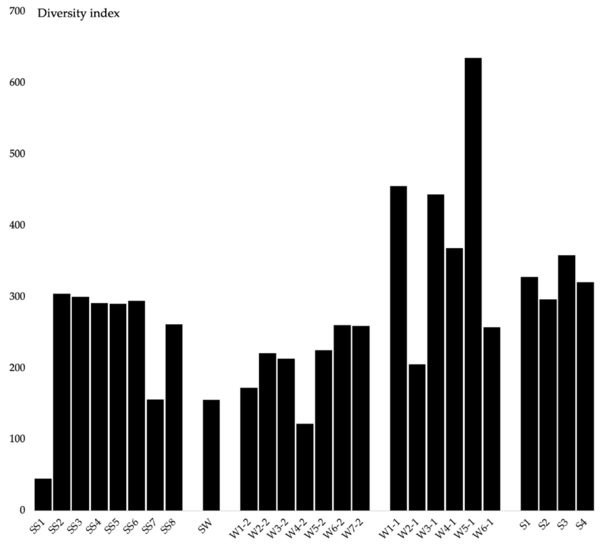

(b)

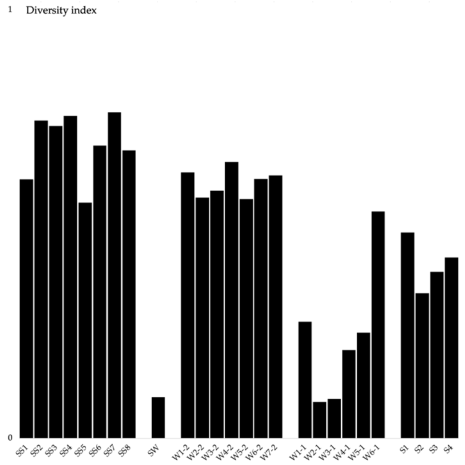

(c)

Figure S1. Alpha-diversity indices for the Bacteria domain, based on (a) the Shannon indices, (b) ASV richness, and (c) evenness. SS, surface soils; S, sediments; W#-1, water 0.1 µm; W#-2, water 0.2 µm; SW, surface water.

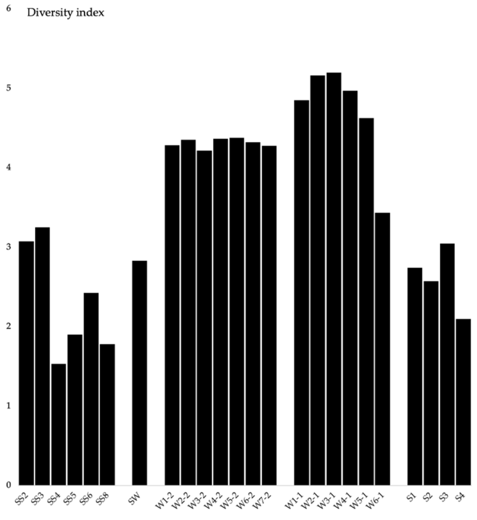

(a)

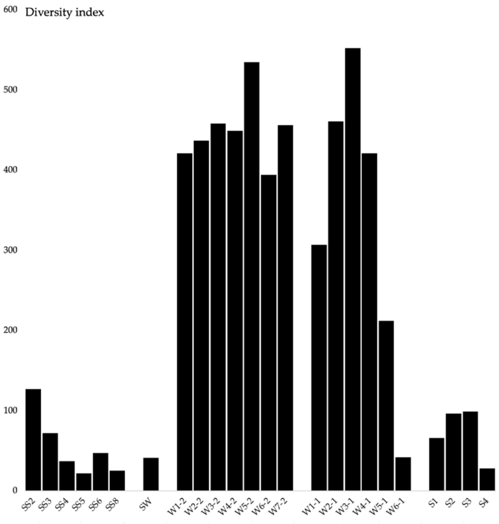

(b)

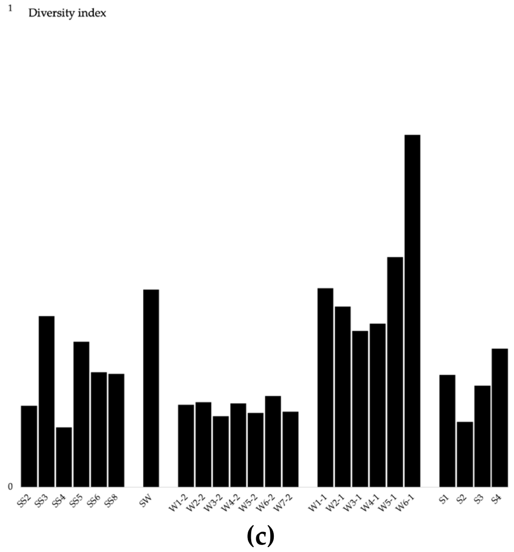

(c)

Figure S2. Alpha-diversity indices for the Archaea domain, based on (a) the Shannon indices, (b) ASV richness, and (c) evenness. SS, surface soils; S, sediments; W#-1, water 0.1 µm; W#-2, water 0.2 µm; SW, surface water.

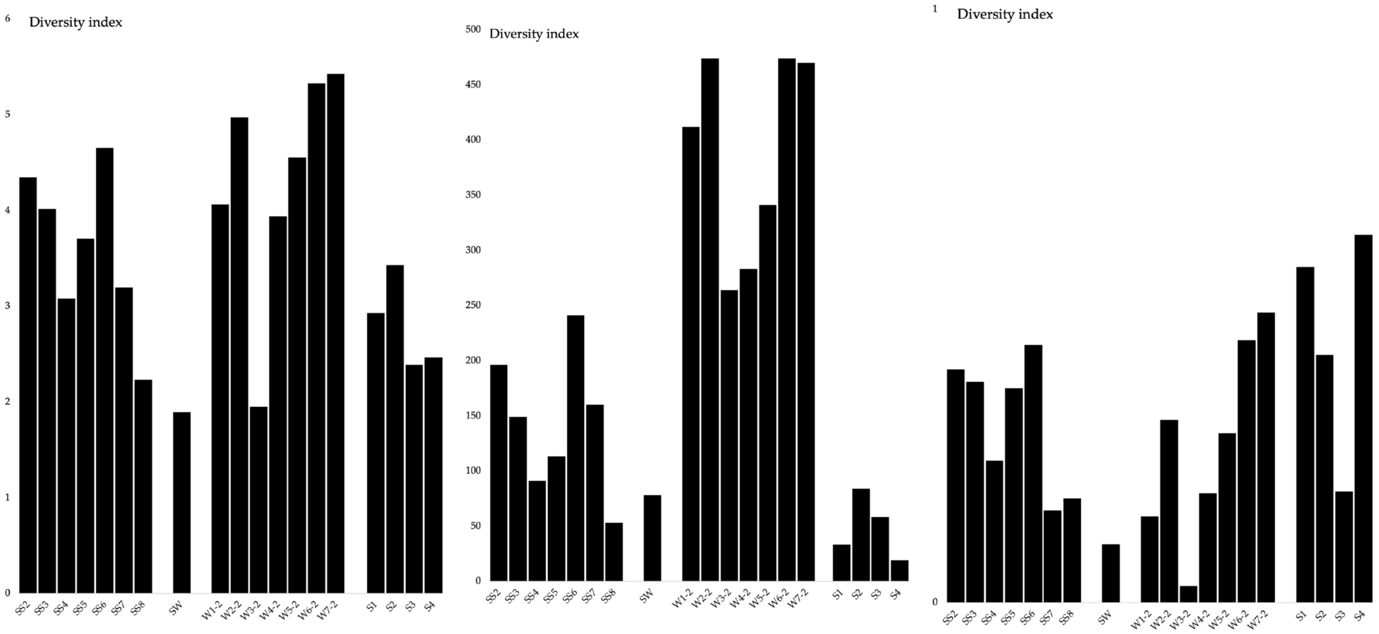

(a) **(b)** **(c)**

Figure S3. Alpha-diversity indices for the Eukaryote domain, based on (a) the Shannon indices, (b) ASV richness, and (c) evenness. SS, surface soils; S, sediments; W#-2, water 0.2 μm; SW, surface water.

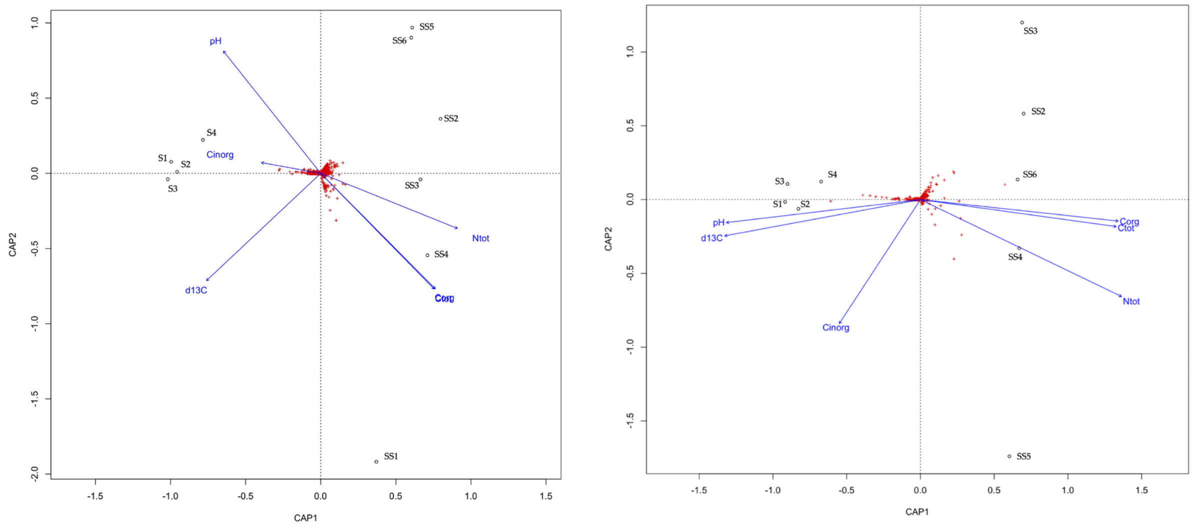

(a) **(b)**

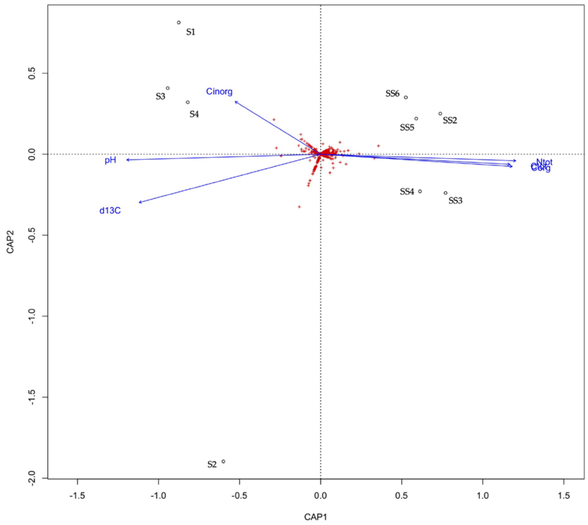

(c)

Figure S4. db-RDA graphs based on a Bray-Curtis dissimilarity matrix for the surface soil/cave sediment communities, for the Bacteria (a), the Archaea (b), and the Eukaryotes (c), and environmental variables measured in each sample. tot, total; org, organic; inorg, inorganic; SoS, SumOfSqs.
